# Priming strategies to enhance the therapeutic efficacy of mesenchymal stromal/stem cell-derived vesicles in regenerative medicine

**DOI:** 10.20517/evcna.2025.84

**Published:** 2026-01-12

**Authors:** Vitale Miceli

**Affiliations:** Department of Research, IRCCS ISMETT (Istituto Mediterraneo per i Trapianti e Terapie ad Alta Specializzazione), Palermo 90127, Italy.

**Keywords:** Mesenchymal stromal/stem cells, extracellular vesicles, priming strategies, regenerative medicine, cell-free therapy

## Abstract

Mesenchymal stromal/stem cell-derived extracellular vesicles (MSC-EVs) have emerged as promising acellular therapeutics in regenerative medicine, offering a safer and more controllable alternative to whole-cell therapies. Their therapeutic efficacy, however, is highly dependent on their molecular cargo, which reflects the physiological state and environmental conditions of the parent MSCs. Priming of mesenchymal stromal/stem cells (MSCs) with defined stimuli such as hypoxia, inflammatory cytokines, 3D culture systems, biomaterials, or pharmacological agents has been increasingly employed to enhance extracellular vesicle (EV) bioactivity. These strategies modulate EV content, enriching vesicles with regenerative, immunomodulatory, angiogenic, and antioxidant factors. For instance, hypoxic priming activates hypoxia-inducible factor-1α-driven gene expression, promoting the packaging of angiogenic and anti-inflammatory molecules, while cytokine-based priming upregulates immunosuppressive proteins and regulatory microRNAs. Similarly, 3D culture mimics aspects of the native tissue microenvironment, augmenting the secretion of EVs with enhanced reparative potential. Emerging combination-based approaches synergize these effects, generating EVs with superior therapeutic profiles. Despite encouraging preclinical data, translation to clinical application is challenged by variability in MSC sources, priming conditions, and EV isolation methods. Standardization of protocols, validated potency assays, and regulatory harmonization are critical for clinical advancement. This mini-review summarizes current priming strategies, the underlying mechanisms influencing EV cargo, and their functional implications in disease models, while highlighting key barriers and future directions for the clinical translation of primed MSC-EV therapies.

## INTRODUCTION

Mesenchymal stromal/stem cells (MSCs) have garnered significant attention in regenerative medicine due to their multifaceted therapeutic properties, such as immunomodulatory, pro-angiogenic, and tissue reparative functions^[1-5]^. While initial clinical applications predominantly utilized whole-cell MSC therapies, concerns regarding potential risks, such as immunogenicity, tumorigenicity, and challenges in storage and delivery, have prompted a paradigm shift toward cell-free therapeutic strategies^[6,7]^. Among these, MSC-derived extracellular vesicles (MSC-EVs), particularly exosomes, have emerged as promising candidates, offering a safer and more controllable alternative that retains the therapeutic efficacy of their parent cells^[1,8,9]^.

MSC-EVs are nano-sized vesicles that encapsulate a diverse array of bioactive molecules, including proteins, lipids, nucleic acids^[10,11]^, and microRNAs (miRNAs)^[12,13]^. The latter belong to the non-coding RNAs (ncRNAs) family and represent one of the most functionally relevant molecular classes within MSC-EV cargo^[14]^. Beyond miRNAs, this heterogeneous group also includes long ncRNAs (lncRNAs), circular RNAs (circRNAs), and piwi-interacting RNAs (piRNAs), which collectively contribute to the epigenetic and post-transcriptional regulation of target genes in recipient cells^[15]^. miRNAs are the most extensively studied members of the ncRNA family and are known to modulate target gene expression, influencing processes such as macrophage polarization/immunomodulation, endothelial cell proliferation, and extracellular matrix remodeling^[12,16-19]^. Thus, miRNA cargo plays a central mechanistic role in the paracrine effects attributed to MSC-EVs and represents a promising target for the rational design of next-generation cell-free therapeutics.

Extracellular vesicles (EVs) play a pivotal role in intercellular communication, modulating various physiological processes such as inflammation, angiogenesis, and tissue regeneration^[10]^. However, the therapeutic potential of MSC-EVs is intrinsically linked to their molecular cargo, which can be influenced by the physiological state, environmental conditions, and source of the parent MSCs^[11,12,20,21]^, as well as the lack of standard operating procedures (SOPs) for large-scale production, which largely depend on quality control and the sources of EVs^[8,22,23]^.

To enhance the therapeutic efficacy of MSC-EVs, various preconditioning or “priming” strategies have been explored^[24-26]^. These include exposing MSCs to hypoxic conditions^[11,27]^, inflammatory cytokines^[16,28]^, three-dimensional (3D) culture systems^[12,25]^, specific biomaterials^[29-32]^, and pharmacological agents^[33]^. Such priming approaches have been shown to modulate the composition of EVs, enriching them with specific miRNAs, proteins, and/or other functional molecules that bolster their regenerative and immunomodulatory functions. For instance, hypoxic preconditioning has been shown to enhance the therapeutic efficacy of EVs by activating hypoxia-inducible factor (HIF-1α), a transcriptional regulator with broad pleiotropic effects. HIF-1α modulates the expression of numerous genes involved in key biological processes, including inflammation, angiogenesis, cell proliferation, differentiation, and apoptosis. This activation also influences the selective enrichment of EV cargo, such as proteins, miRNAs, and growth factors, which contribute to the regulation of regenerative mechanisms, both under physiological and pathological conditions^[34]^. Similarly, inflammatory cytokine priming can increase the expression of immunosuppressive molecules, as well as a plethora of miRNAs with immunomodulatory abilities within EVs, augmenting their capacity to modulate immune responses^[12,16,35]^. Moreover, 3D culture has emerged as an effective priming strategy capable of enhancing the regenerative potential of MSC-EVs, by enriching both their protein^[36]^ and miRNA^[12]^ content.

Despite these advancements, challenges persist in standardizing priming protocols and understanding the precise mechanisms by which different priming conditions influence EV composition and function. Moreover, the heterogeneity of MSC sources and the variability in EV isolation and characterization methods further complicate the translation of these findings into clinical applications. This review aims to provide an overview of current priming strategies employed to enhance the therapeutic potential of MSC-EVs. It discusses the underlying mechanisms by which priming influences EV cargo, evaluates the functional outcomes in various disease models, and highlights the challenges and future directions in the clinical translation of primed MSC-EV therapies.

## MSC-DERIVED VESICLES: BIOLOGY AND THERAPEUTIC MECHANISMS

MSC-EVs, including exosomes (30-150 nm in size)^[37]^, constitute a key functional component of the MSC secretome and act as potent mediators of intercellular communication^[10,22,38]^. These nanoscale vesicles encapsulate a heterogeneous array of bioactive molecules, such as miRNAs, proteins, lipids, and growth factors^[10-13]^, whose composition is dynamically influenced by both the tissue origin of the MSCs (e.g., bone marrow, umbilical cord, adipose tissue)^[20]^ and external environmental conditions including hypoxia, inflammation, and 3D culture systems^[11,12,21]^. Their biogenesis follows both endosomal sorting complexes required for transport (ESCRT)-dependent and ESCRT-independent pathways, reflecting a tightly regulated packaging mechanism that governs cargo specificity and biological functionality^[39]^.

Functionally, MSC-EVs exhibit remarkable therapeutic plasticity across five major pathophysiological processes. First, their immunomodulatory activity can be mediated by the delivery of immunosuppressive molecules such as indoleamine 2,3-dioxygenase (IDO), interleukin (IL)-10, and human leukocyte antigen class I molecule G (HLA-G), along with miRNAs including miR-21, miR-181c, miR-155, and miR-146a. These molecules collectively suppress pro-inflammatory signaling, promote polarization of macrophages toward the M2 phenotype (alternatively activated cells), and enhance regulatory T-cell responses^[35]^. Second, MSC-EVs can exert pro-angiogenic and regenerative effects through factors such as vascular endothelial growth factor (VEGF), fibroblast growth factor (FGF)-2, miR-21, and miR-221, which activate endothelial receptors [e.g., VEGF receptor (VEGFR)] to support neovascularization, perfusion, and structural repair in ischemic models^[40]^. Third, their anti-fibrotic properties emerge from the inhibition of transforming growth factor (TGF)-β/Smad and Wnt/β-catenin pathways, along with the modulation of matrix metalloproteases, limiting excessive extracellular matrix deposition in fibrotic tissue environments^[41,42]^. Fourth, neuroprotective functions are mediated through EV-associated miRNAs that support neuronal survival, axonal growth, and neurogenesis following ischemic or traumatic central nervous system injury^[43]^. Fifth, the antioxidant capacity of MSC-EVs, an increasingly recognized feature, can be mediated via antioxidant enzymes such as catalase and superoxide dismutase^[44]^, as well as redox-modulating miRNAs^[17,45]^ - which reduce reactive oxygen species (ROS) accumulation, attenuate oxidative stress, and limit cellular damage across various disease contexts^[46]^.

Importantly, the therapeutic efficacy of MSC-EVs in all these processes can be significantly modulated through priming of the parental MSCs. Strategies such as hypoxic exposure, cytokine stimulation, 3D culture, and combinations of these strategies have been shown to enhance the functional properties of EVs by enriching their cargo with regenerative, immunomodulatory, angiogenic, and antioxidant molecules^[33]^.

Recent early-phase preclinical and clinical studies have begun to evaluate the safety and therapeutic efficacy of MSC-EVs in a range of conditions, including neurodegenerative disorders, ischemic injuries, immune-related diseases, and tissue degeneration disorders^[47-49]^. Nevertheless, key challenges remain in establishing standardized protocols for EV production, isolation, characterization, potency assessment, and dosage determination, all of which are critical to fully realize their translational and clinical potential^[50]^.

## THE CONCEPT AND PURPOSE OF PRIMING

Priming, also referred to as preconditioning or licensing, is an *in vitro* strategy designed to modulate the functional behavior of MSCs by exposing them to specific stimuli, including hypoxia, inflammatory cytokines, or 3D culture systems. This approach aims to enhance the therapeutic efficacy of both MSCs and their products, including EVs^[11,12,16,24-26,51,52]^. It is based on the growing understanding that the MSC microenvironment critically influences their paracrine profile, including EV composition, which in turn governs functional outcomes in recipient cells and tissues.

In contrast to genetic engineering techniques, priming offers a non-integrative, transient, and potentially safer means to fine-tune EV content. Through the selective enrichment of vesicular proteins, miRNAs, enzymes, and other regulatory molecules, priming can direct EV function toward specific pathophysiological pathways. This not only improves therapeutic precision and reproducibility but also facilitates regulatory compliance and enhances the overall biosafety profile of MSC-based products. Notably, combination-based strategies of priming, which integrate multiple stimuli, have demonstrated synergistic effects, resulting in EVs with significantly enhanced therapeutic properties^[53]^.

Currently, despite promising results in various preclinical models^[54-58]^, the lack of standardized priming protocols remains a major translational bottleneck. Critical parameters, including stimulus type, concentration, duration, MSC source, and subsequent EV isolation and characterization, must be harmonized to ensure reproducibility, batch consistency, and alignment with Good Manufacturing Practice (GMP) requirements. Priming represents a pivotal translational bridge, positioned between basic research and clinical application, guiding the development of MSC-EV-based therapies toward more precisely engineered, disease-specific, and clinically validated interventions in regenerative medicine.

## PRIMING STRATEGIES: EXPERIMENTAL EVIDENCE AND MECHANISTIC INSIGHTS

Recent preclinical and omics studies^[11,12,16,24-26,51-53,59,60]^ have delineated how diverse priming strategies, including exposure of MSCs to specific biomaterials, inflammatory cytokines or other molecules, 3D culture, and combination-based strategies, modulate MSC-EV cargo and significantly enhance their therapeutic activities [Table 1].

**Table 1:** Summary of MSC priming strategies, EV cargo modulation, and associated therapeutic applications

**Priming strategy**	**Representative EV cargo modulation (miRNAs/proteins)**	**Main functional effects**	**Disease models/therapeutic contexts**	**Key References**
Hypoxia	Increased miR-126, mmu_circ_0001295, miR-612, VEGFA-associated signaling molecules	Enhanced angiogenesis and tissue regeneration; reduced sepsis-induced renal injury	Myocardial infarction; ischemic tissue repair; renal injury	[63-66]
Inflammatory cytokine priming (e.g., IFN-γ, IL-1β, LPS)	Increased miR-21, let-7b, PD-L1, TGF-β1	Immunomodulation and macrophage polarization toward pro-repair (M2-like) phenotypes	Autoimmune inflammation, sepsis, GvHD	[58,60,69,70]
3D spheroid culture	Increased let-7 family, miR-132, pro-angiogenic proteins (HGF, VEGF)	Enhanced pro-regenerative paracrine signaling and ECM remodeling	Acute kidney injury, cardiac repair	[54,71,73,74]
Biomaterial-mediated priming (e.g., tailored stiffness, RGD presentation)	Selective enrichment of osteo- or chondro-regenerative miRNAs (miR-221/222); modulators of integrin-YAP/TAZ signaling	Directed lineage specification; improved matrix remodeling and regeneration; immunomodulation	Osteochondral regeneration; bone defect healing; skin injury models	[31,32,78,81,83]
Combined priming (e.g., 3D + hypoxia/cytokines)	Coordinated increase in BMP2/7, BDNF, NGF, VEGF, IDO1, IL-10	Synergistic pro-repair, pro-angiogenic, and anti-inflammatory effects	Complex inflammatory/ischemic tissue damage	[53,86,87]

MSC: Mesenchymal stem cell; EV: extracellular vesicle; miRNA: microRNA; VEGFA: vascular endothelial growth factor A; IFN-γ: interferon gamma; IL-1β: interleukin 1 beta; IL-10: interleukin 10; LPS: lipopolysaccharide; PD-L1: programmed death-ligand 1; TGF-β1: transforming growth factor beta 1; ECM: extracellular matrix; GvHD: graft-versus-host disease; HGF: hepatocyte growth factor; VEGF: vascular endothelial growth factor; RGD: arginine-glycine-aspartic acid; YAP: Yes-associated protein; TAZ: transcriptional coactivator with PDZ-binding motif; BMP2/7: bone morphogenetic protein 2/7; BDNF: brain-derived neurotrophic factor; NGF: nerve growth factor; IDO1: indoleamine 2,3-dioxygenase 1.

Hypoxic conditioning stabilizes HIF-1α in MSCs, resulting in EVs enriched with angiogenic factors^[61,62]^. Hypoxia has emerged as a potent strategy to enhance MSC-EV regenerative potential, particularly in promoting angiogenesis^[62]^. Consistent with this, Cao *et al*. demonstrated that intravenous administration of EVs from hypoxia-primed MSCs reduced sepsis-induced renal injury in mice, mediated by high levels of circRNA mmu_circ_0001295^[63]^. Hypoxia-primed EVs also improve angiogenesis in cerebral ischemia models, attributed to proteins such as vascular endothelial growth factor A (VEGFA), hepatocyte growth factor (HGF), chemokine (C-C motif) ligand 11 (EOTAXIN), angiopoietin-like 4 (ANGPTL4), insulin-like growth factor-binding protein 3 (IGFBP3), and EGF-like repeat and discoidin I-like domain-containing protein 3 (EDIL3), as well as miRNAs such as miR-126 and miR-612^[11,64-66]^. Additionally, hypoxia enhances both EV bioactivity and secretion^[62]^. Similarly, dimethyloxaloylglycine (DMOG), a HIF-1α stabilizer, augments MSC-EV pro-angiogenic properties through activation of the protein kinase B (AKT)/mammalian target of rapamycin (mTOR) pathway^[67]^. Hypoxia-primed EVs also demonstrate enhanced immunosuppressive effects, notably promoting M2 polarization of microglia and macrophages in Alzheimer’s disease models^[68]^.

Inflammatory priming has also demonstrated benefits in improving MSC-EV efficacy. Recently, it has been shown that interferon gamma (IFN-γ) priming, unlike hypoxia exposure, induces an immunosuppressive EV profile enriched in TGF-β1, annexin A1 (ANXA1), and thrombospondin 1 (THBS1). In contrast, hypoxic priming generates a pro-angiogenic, tissue-regenerative phenotype, characterized by elevated VEGFA, platelet-derived growth factor receptor beta (PDGFRB), ANGPTL4, endoglin (ENG), growth-regulated oncogene gamma (GRO-γ), IL-8, and GRO-α. Interestingly, these phenotypes were functionally validated^[11]^. IFN-γ treatment was able to upregulate EV miRNAs such as miR-452, miR-125b, miR-9, miR-23a, miR-26b, miR-33b, miR-130b, miR-133b, and miR-139 - which can regulate chronic inflammation, T-cell activation/anergy, monocyte differentiation, and cytokine signaling^[12,16]^. Lipopolysaccharide (LPS)-stimulated MSCs produced EVs that attenuated inflammation and promoted wound healing in diabetic rats via let-7b-mediated M2 polarization^[69]^. IL-1β-primed MSC-EVs exhibited greater anti-inflammatory activity in osteoarthritic SW982 cells, attributed to miR-147b enrichment^[70]^, while miR-21 in IL-1β-primed EVs facilitated macrophage M2 polarization and improved outcomes in sepsis^[58]^. A combination of inflammatory cytokines (IFN-γ, TNF-α, IL-1β) further enhanced immunoregulatory function; triple-primed EVs showed improved outcomes in graft-versus-host disease (GvHD), partially due to programmed death-ligand 1 (PD-L1) upregulation in the EVs^[60]^.

Three-dimensional culture systems, including microcarriers and spheroids grown in bioreactors, more closely mimic physiological conditions and produce EVs enriched in angiogenic and immunomodulatory cargo^[12,71,72]^. These EVs carry elevated levels of miRNAs (let-7a/c, miR-13b, miR-132, miR-200c, miR-212, miR-214, miR-21, miR-22, miR-34a/b)^[12]^ and proteins [collagen type I alpha 1 chain (COL1A1), FGF, VEGF, epidermal growth factor (EGF), HGF, IL-11, TGF-β1, THBS1]^[73]^. Recently, it has been shown that 3D-primed EVs displayed improved therapeutic efficacy in myocardial infarction and cisplatin-induced acute kidney injury models^[54,74]^. Further enhancement was observed when combining 3D culture with TNF-α/IFN-γ priming, yielding EVs with superior regenerative potential^[75]^.

Oxidative and sulfide-based priming represents another promising strategy. These agents modulate the expression and selective packaging of EV-associated miRNAs, thereby enhancing the vesicles’ antioxidant, anti-inflammatory, and cytoprotective functions^[33]^. For instance, hydrogen sulfide (H_2_S) upregulated miR-7b in MSC-EVs, promoting anti-inflammatory and neuroprotective effects on microglia and monocyte-derived macrophages^[76]^. Nitric oxide (NO) priming elevated VEGF and miR-126 in EVs from placenta-derived MSCs, enhancing endothelial proliferation and angiogenesis in ischemic models^[77]^.

In addition to biochemical and culture-based priming approaches, biomaterial-based strategies have emerged as a critical and rapidly advancing method in the engineering of MSC-EVs. A growing body of mechanobiology research has demonstrated that extracellular matrix-mimetic cues, including substrate stiffness, surface topography, and biochemical ligand presentation, can strongly influence MSC phenotype, intracellular signaling, and downstream EV biogenesis and cargo loading. For instance, MSCs cultured on matrices with defined mechanical stiffness exhibit differential yes-associated protein (YAP)/transcriptional co-activator with PDZ-binding motif (TAZ) activation, cytoskeletal remodeling, and lineage-specific gene expression^[78,79]^. Emerging evidence suggests that such mechanobiological cues can also influence the molecular composition of MSC-EVs, with corresponding shifts in EV-associated miRNAs and proteins that regulate cartilage homeostasis and subchondral bone remodeling^[30,32]^. Likewise, cultivation on nanopatterned or fibrous scaffolds has been shown to enhance the secretion of EVs enriched in regenerative factors capable of promoting osteogenesis, vascularization, matrix remodeling, and macrophage polarization toward pro-repair phenotypes^[31,80-82]^. Furthermore, the functionalization of biomaterials with cell-adhesive peptides [e.g., arginine-glycine-aspartic acid (RGD) motifs] or growth factor-mimetic ligands can further fine-tune paracrine activity, resulting in EVs with enhanced immunomodulatory properties^[29,83]^. Importantly, these biomaterial-mediated priming approaches are highly tunable, allowing precise adjustment of physicochemical parameters to direct EV cargo composition, while also being compatible with scalable manufacturing platforms such as dynamic bioreactors and microcarrier-based systems^[84,85]^. As such, the integration of materials science and mechanotransduction principles provides a versatile and powerful means to improve the therapeutic efficacy, reproducibility, and translational readiness of MSC-EV products, complementing existing biochemical priming strategies and expanding the conceptual framework of EV engineering in regenerative medicine. Moreover, combination-based strategies of priming, incorporating 3D culture, hypoxia, and inflammatory stimuli, further enhance EV cargo complexity, synergistically increasing osteogenic [bone morphogenetic protein (BMP) 2/7], neurotrophic [brain-derived neurotrophic factor (BDNF), nerve growth factor (NGF)], angiogenic (VEGF), and immunomodulatory [Indoleamine 2,3-Dioxygenase 1 (IDO1), IL-10] factors. RNA sequencing and proteomic analyses confirm that these EVs possess greater stability and therapeutic potency^[53,86]^. Mechanistically, priming influences key signaling pathways, Janus kinase (JAK)/signal transducer and activator of transcription (STAT) and phosphatidylinositol-4,5-bisphosphate 3-kinase (PI3K)/AKT (via IFN-γ, TNF-α), as well as HIF-1α/VEGFR (via hypoxia), to reshape EV composition^[87]^.

Together, this scientific evidence demonstrates that targeted priming enhances MSC-EV efficacy across immunomodulatory, angiogenic, neuroprotective, and regenerative processes, providing mechanistic foundations that support their clinical translation.

## CONSIDERATIONS FOR CLINICAL TRANSLATION

Despite the expanding body of evidence supporting priming strategies to enhance the therapeutic profile of MSC-EVs, significant challenges remain in translating these findings to clinical practice. First, standardization of EV production is crucial. Variations in MSC source, culture conditions, priming stimuli (type, dose, and duration), and EV isolation methods introduce heterogeneity that hampers reproducibility and regulatory compliance^[88]^. Establishing standardized protocols aligned with GMP guidelines is imperative to ensure batch-to-batch consistency and product safety. Robust and scalable manufacturing platforms must be developed for large-scale EV production, and quality control parameters, including particle size distribution, surface marker profiling, and functional potency assays, require harmonization across laboratories and clinical trials. Critical is the development of validated potency assays that can predict clinical efficacy. In fact, while current approaches often rely on *in vitro* surrogate markers (e.g., macrophage polarization, endothelial tube formation)^[89,90]^, more sophisticated functional assays and biomarker-based readouts are needed to establish predictive correlations with therapeutic outcomes. Furthermore, issues related to storage stability, dosing regimens, delivery routes (e.g., systemic *vs*. local), and pharmacokinetics remain inadequately defined^[91,92]^ and must be addressed through rigorously designed preclinical studies and early-phase clinical trials.

From a regulatory perspective, EVs currently reside in a complex space between biologics and drug products, raising questions about classification, characterization requirements, and licensing pathways. Regulatory approval of EV-based therapeutics requires compliance with the Food and Drug Administration (FDA) framework for biological products, which emphasizes product characterization, reproducibility, and clinical safety^[93]^, and must also align with Minimal Information for Studies of Extracellular Vesicles (MISEV)/International Society for Extracellular Vesicles (ISEV) guidelines^[93,94]^. A key element is the clear definition of the cell source used to generate EVs. Primary MSCs derived from different sources can differ substantially in proliferative capacity, immunomodulatory phenotype, and secretory profiles^[20,95]^. These intrinsic differences highlight the need for source-specific qualification criteria, including standardized donor eligibility and predefined passage-number thresholds, to avoid senescence-associated changes in EV cargo composition^[93,94]^. In parallel, induced pluripotent stem cell-derived MSCs are increasingly recognized as a promising alternative due to their capacity for renewable, scalable production under controlled differentiation protocols, reducing donor-dependent variability^[47]^. Finally, EV production must be conducted under GMP conditions, with standardized culture systems, reproducible EV isolation and purification workflows, and potency assays that correlate the biological mechanism with the intended therapeutic effect^[93,94]^. These elements collectively ensure that the EV product can be manufactured consistently at scale, and meet both FDA and MISEV/ISEV guidelines for studies and subsequent clinical trial evaluations. Thus, a collaborative effort involving regulatory agencies, academia, and industry is essential to define appropriate regulatory frameworks, enabling the safe and effective clinical translation of primed MSC-EV therapies.

## CONCLUSIONS AND FUTURE PERSPECTIVES

**Figure 1:**
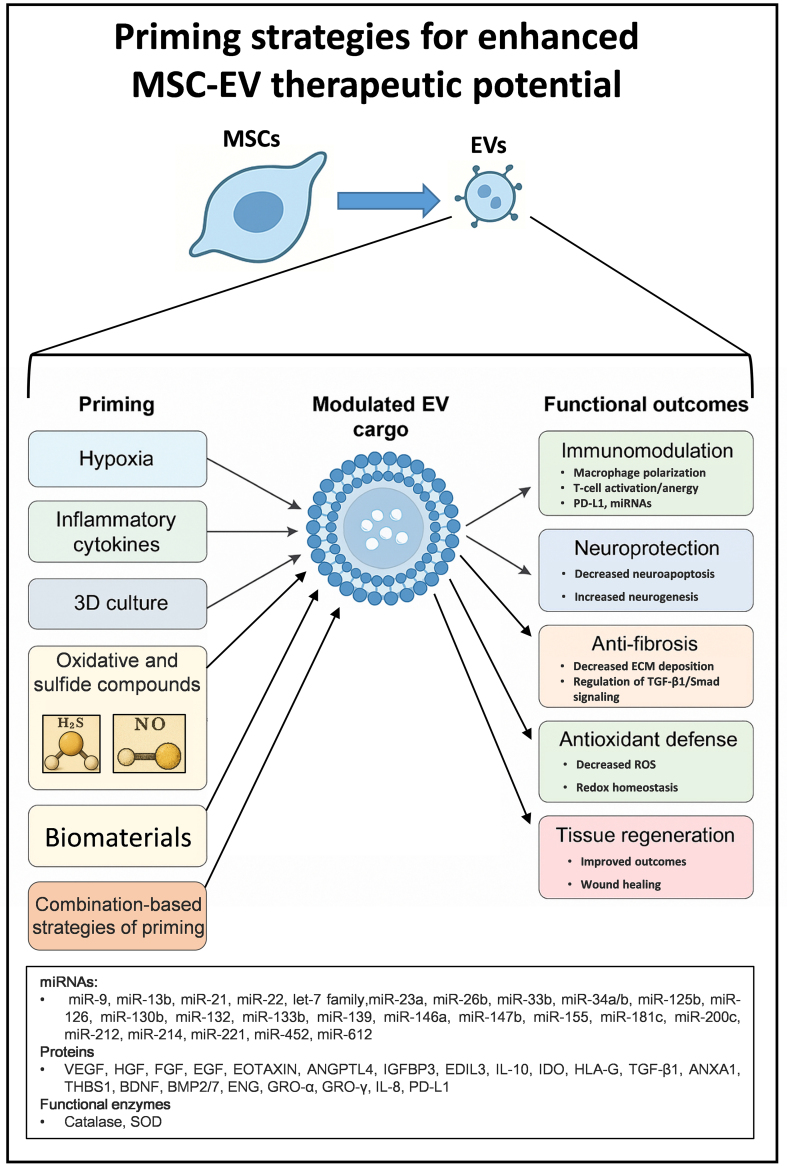
Schematic overview of MSC priming strategies and EV functional modulation. This diagram illustrates the principal priming strategies applied to MSCs, including hypoxia, inflammatory cytokines, 3D culture systems, oxidative and sulfide compounds, and biomaterials. These stimuli influence the MSC secretory profile, leading to the production of EVs enriched in regulatory microRNAs, proteins and antioxidant enzymes. The molecular reprogramming of EV cargo enhances their therapeutic potential, conferring immunomodulatory, angiogenic, neuroprotective, anti-fibrotic, and antioxidant properties^[11,12,16,24-26,30-33,51-54,58-62,64-87]^. These features can collectively promote tissue repair and regeneration across diverse disease models. MSC: Mesenchymal stem cell; EV: extracellular vesicle; 3D: three-dimensional; H_2_S: hydrogen sulfide; NO: nitric oxide; ECM: extracellular matrix; TGF-β1: transforming growth factor beta 1; ROS: reactive oxygen species; PD-L1: programmed death-ligand 1; miRNA: microRNA; VEGF: vascular endothelial growth factor; HGF: hepatocyte growth factor; FGF: fibroblast growth factor; EGF: epidermal growth factor; ANGPTL4: angiopoietin-like 4; IGFBP3: insulin-like growth factor binding protein 3; EDIL3: EGF-like repeats and discoidin I-like domains 3; IL: interleukin; IDO: indoleamine 2,3-dioxygenase; HLA-G: human leukocyte antigen G; THBS1: thrombospondin 1; BDNF: brain-derived neurotrophic factor; BMP2/7: bone morphogenetic protein 2/7; ENG: endoglin; GRO-α: growth-regulated oncogene alpha; GRO-γ: growth-regulated oncogene gamma; SOD: superoxide dismutase.

Priming represents an innovative strategy with promising therapeutic implications in the field of MSC-EV-based therapies, enabling the customization of EV cargo to maximize therapeutic outcomes. By recapitulating key aspects of the MSC microenvironment, such as hypoxia, inflammatory or oxidative stimuli, and 3D culture, priming enhances the functional potency of EVs across critical biological processes, including immunomodulation, tissue regeneration, neuroprotection, fibrosis and oxidative stress mitigation [Figure 1].

Emerging data demonstrate that primed EVs consistently outperform unprimed counterparts in preclinical models of tissue injury, immune dysregulation, and fibrosis. Furthermore, combination-based strategies of priming have shown synergistic effects, generating EVs with enhanced complexity and multi-target functionality^[96-99]^. This multimodal strategy offers a refined, pathology-specific platform for generating next-generation cell-free therapeutics with superior potency and translational relevance.

An additional promising avenue for advancing the therapeutic potency of MSC-EVs lies in the use of MSC-based organoid systems^[100]^. Unlike conventional 3D culture or spheroid aggregation, organoids recreate key architectural and functional features of native tissue microenvironments, including multi-lineage cellular organization, spatially regulated extracellular matrix deposition, and dynamic cell-cell and cell-matrix signaling^[100]^. Within these niches, MSCs can be exposed to instructive biochemical gradients and mechanical cues that are difficult to reproduce in standard culture conditions, potentially resulting in more physiologically relevant EV biogenesis and selective cargo loading. Therefore, EVs derived from MSC-organoid systems could exhibit a more coordinated and integrated molecular profile, reflecting the complex reparative signaling networks characteristic of developing or regenerating tissues. This approach holds particular promise for enhancing EV-mediated tissue remodeling, angiogenesis, immunoregulation, and metabolic support in disease settings. As such, the integration of organoid biology into EV manufacturing platforms could represent a natural progression toward next-generation acellular therapeutics, and merits dedicated investigation in future translational studies.

However, clinical translation remains contingent upon the resolution of several technical and regulatory barriers. Future research should prioritize the optimization of scalable manufacturing systems, the identification of predictive potency markers, and the establishment of standardized protocols. Moreover, integrating high-throughput omics with machine learning approaches may further elucidate EV cargo-function relationships, enabling the rational design of precision vesicle therapies.

Ultimately, primed MSC-EVs stand at the frontier of regenerative medicine, offering a promising and adaptable therapeutic platform. With sustained interdisciplinary collaboration, these vesicles may soon bridge the gap between laboratory discovery and clinical application, delivering targeted, safe, and effective solutions for complex diseases.

## DECLARATIONS

### Copyright

© The Author(s) 2026.
